# The Complete Anatomy of the Anterior Ethmoidal Artery: A Meta-Analysis with Implications for Sinus and Skull Base Surgery

**DOI:** 10.3390/jcm13061695

**Published:** 2024-03-15

**Authors:** Elżbieta Szczepanek, Julia Toppich, Patryk Ostrowski, Michał Bonczar, Ameen Nasser, Martyna Dziedzic, Jerzy Walocha, Mateusz Koziej

**Affiliations:** 1 Department of Anatomy, Jagiellonian University Medical College Cracow, 31-034 Kraków, Poland; 2 Doctoral School in Medical Sciences and Health Sciences, Jagiellonian University Medical College, 31-008 Kraków, Poland; 3 Youthoria, Youth Research Organization, 31-127 Kraków, Poland

**Keywords:** anterior ethmoidal artery, skull base, sinus, surgery, anatomy

## Abstract

**Background:** The anatomical characteristics of the anterior ethmoidal artery (AEA) exhibit a high degree of variability, especially regarding its topography. **Methods:** PubMed, Scopus, Embase, Web of Science, Cochrane Library, and Google Scholar were searched to identify all studies that included information regarding the morphometric and topographical characteristics of the AEA. **Results:** Ultimately, a compilation of 38 studies meeting the predetermined criteria, and possessing comprehensive and pertinent data, were incorporated into the current meta-analysis. In the overall analysis, reports of the AEA being embedded within the skull base or at the level of the skull base producing a bony protrusion were found in 56.91% of cases (95% CI: 43.55–69.80%). The mean diameters of the AEA in the intraorbital and intracranial areas were 0.94 mm and 0.76 mm, respectively. Moreover, the artery coursed most commonly between the second and third lamellae, with a prevalence of 65.82% (95% CI: 55.39–75.56%). **Conclusions:** The AEA may be at a high risk of iatrogenic injury during various sinus and skull base surgeries, especially if the surgeon performing these procedures is unfamiliar with the vast number of variations this vessel may exhibit. The spatial relationship between this vessel and the skull base is especially variable, and understanding this variability goes hand in hand with intraoperative safety.

## 1. Introduction

**Figure 1:**
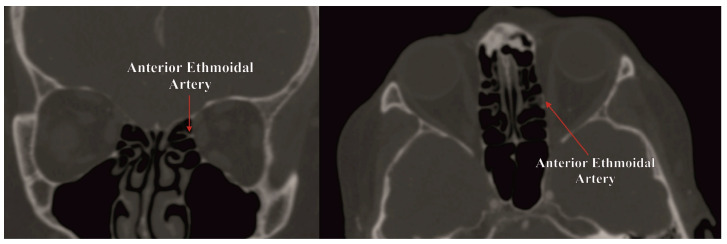
Anterior ethmoidal artery observed through computed tomography angiography—coronal and axial views.

**Figure 2:**
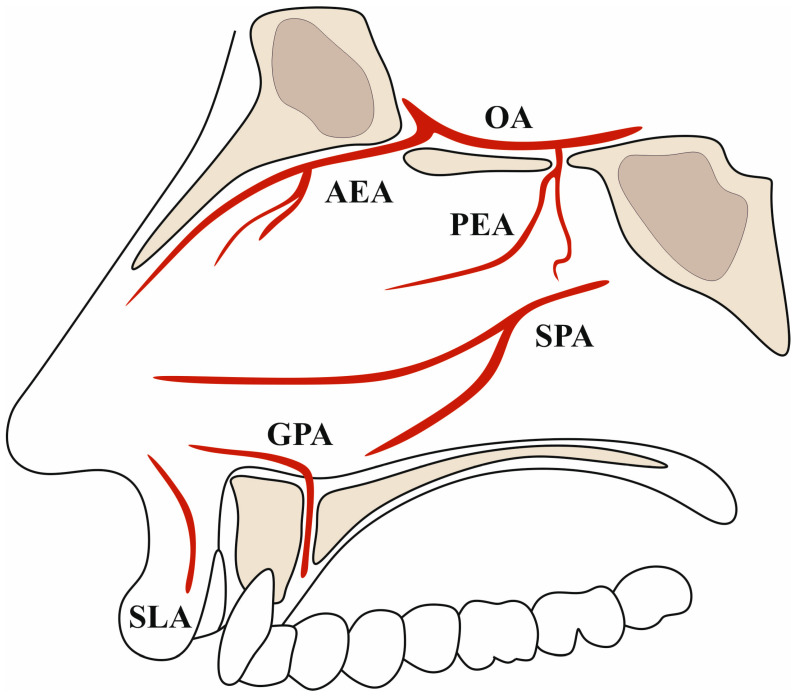
Illustration presenting the vascular supply of the nasal septum. AEA—anterior ethmoidal artery. OA—ophthalmic artery. PEA—posterior ethmoidal artery. SPA—sphenopalatine artery. GPA—greater palatine artery. SLA—superior labial artery.

The anterior ethmoidal artery (AEA) arises from the ophthalmic artery distal to the point of origin of the posterior ethmoidal artery. It exits the medial orbital wall through the anterior ethmoidal foramen, subsequently crossing the anterior ethmoid air cells through the anterior ethmoidal canal to the anterior cranial fossa (Figure 1). The AEA produces branches both in the anterior ethmoidal canal (extracranially) and after emerging from the anterior ethmoidal foramen (intracranially). The extracranial branches consist of arteries supplying the nasal cavity and septum, as well as the anterior ethmoidal cells and the frontal sinus (Figure 2). On the other hand, the intracranial branches, such as the anterior falcine artery, supply the meninges of the anterior cranial fossa [1,2].

The topography of the AEA exhibits a high degree of variability in the ethmoid sinus and the skull base region [3,4]. Previous studies have described the spatial relationship between the AEA and the skull base using a classification system consisting of three types: Type A represents the artery embedded in the skull base. Next, Type B presents the AEA coursing at the level of the skull base, creating a bony protrusion. Finally, Type C refers to the vessel coursing freely in the ethmoid sinus while being connected to the skull base by a thin bony mesentery [5,6,7]. However, there are big discrepancies regarding the overall prevalence of the abovementioned types in the literature, with some studies reporting the AEA to course most commonly within the skull base (Type A) [8,9,10], while others state that the vessel is located most commonly below it (Type C) [5,11]. The relationship between the AEA and the four lamellae, i.e., the vertical bony structures that compartmentalize the ethmoid sinus, has also been analyzed by numerous researchers in the past [12,13]. A deep understanding of the topography of the AEA and its spatial relationships to nearby landmarks is of utmost importance during endoscopic sinus surgery (ESS) and endoscopic skull base surgery (ESBS) because iatrogenic injury to this vessel may lead to detrimental complications, such as cerebrospinal fluid leak, retro-orbital hematoma, and vision loss [14,15,16]. Hence, the objective of this study was to provide surgeons with the most current and evidence-based information concerning the anatomy of the AEA. Our results may aid in increasing the efficiency and safety of intraoperative localization of this vessel during surgeries in the craniofacial region.

## 2. Materials and Methods

### 2.1. Search Strategy

To conduct this meta-analysis, a systematic search was carried out, targeting all articles that aimed to establish the anatomy of the AEA. The investigation involved thorough searches on prominent online medical databases, including PubMed, Scopus, Embase, Web of Science, Google Scholar, and Cochrane Library. The search procedure was carried out in three phases. (1) Initially, all of the aforementioned medical databases were explored using the specific search term “anterior ethmoidal artery”, without any restrictions regarding the article’s date, language, type, or text availability. (2) Moreover, the specified databases underwent a secondary search employing a different set of search terms: (a) (anterior ethmoidal artery [Title/Abstract]) AND (anatomy [Title/Abstract]); (b) (anterior ethmoidal artery [Title/Abstract]) AND (type [Title/Abstract]); (c) (anterior ethmoidal artery [Title/Abstract]) AND (variant [Title/Abstract]); (d) (anterior ethmoidal artery [Title/Abstract]) AND (topography [Title/Abstract]); (e) (anterior ethmoidal artery [Title/Abstract]) AND (morphology [Title/Abstract]). (3) Subsequently, an extra manual search was conducted across all references cited in the initially submitted studies. The Preferred Reporting Items for Systematic Reviews and Meta-Analyses (PRISMA) guidelines were followed. Additionally, the Critical Appraisal Tool for Anatomical Meta-Analysis (CATAM) and Anatomical Quality Assessment Tool (AQUA) were used to provide the highest-quality findings [17,18].

### 2.2. Eligibility Assessment and Data Extraction

The criteria for inclusion were outlined as follows: original articles containing retrievable information concerning the anatomy, morphology, location, or variations of the AEA. Exclusion criteria comprised conference abstracts, individual case reports, case series, reviews, letters to the editor, and studies lacking pertinent or compatible data. The systematic search was carried out independently by two authors.

Two independent researchers extracted data from eligible studies. Qualitative data, including the year of publication and the country and continent of origin, were gathered. Subsequently, quantitative data regarding the AEA’s diameter, length, types, and localization were extracted. Any disparities found between the studies identified by the two researchers were addressed either by reaching out to the authors of the original studies, when feasible, or by reaching a consensus with a third researcher.

### 2.3. Statistical Analysis

To perform this meta-analysis, STATISTICA version 13.1 software (StatSoft Inc., Tulsa, OK, USA), MetaXL version 5.3 software (EpiGear International Pty Ltd., Wilston, QLD, Australia), and Comprehensive Meta-Analysis version 4.0 software (Biostat Inc., Englewood, NJ, USA) were used. A random effects model was used. The Chi-square test and the I-squared statistic were chosen to assess the heterogeneity among the studies [19]. *p*-values and confidence intervals were used to determine the statistical significance between the studies. A *p*-value lower than 0.05 was considered statistically significant. In the event of overlapping confidence intervals, the differences were considered statistically insignificant. I-squared statistics were interpreted as follows: values of 0–40% were considered as “might not be important”, values of 30–60% were considered as “might indicate moderate heterogeneity”, values of 50–90% were considered as “may indicate substantial heterogeneity”, and values of 75–100% were considered as “may indicate substantial heterogeneity”.

## 3. Results

**Figure 3:**
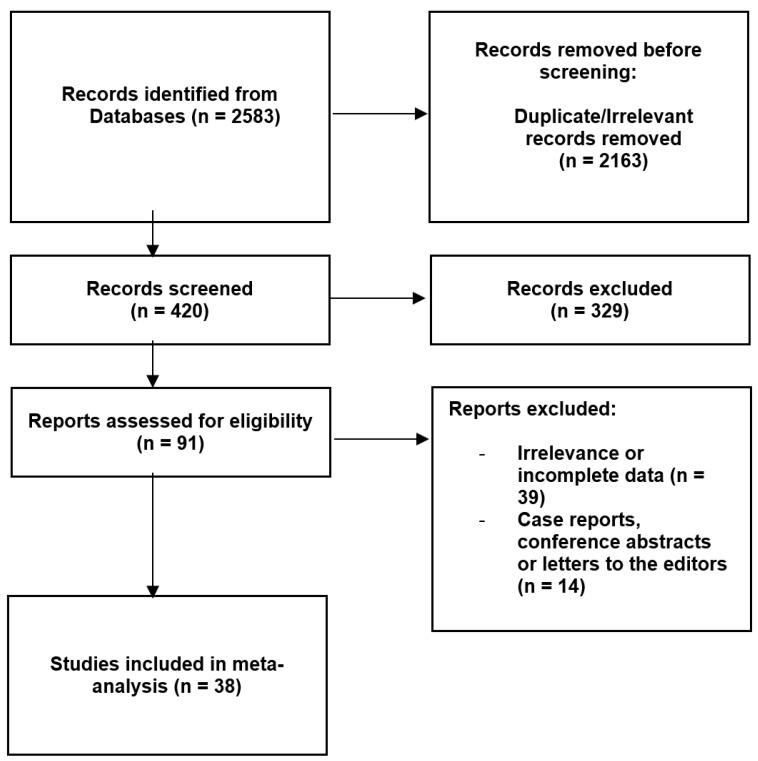
Flow diagram presenting the process of collecting the data included in this meta-analysis.

Initially, 2583 articles underwent evaluation. Ultimately, 38 articles met the required criteria and were included in this meta-analysis [4,5,6,7,8,9,10,11,20,21,22,23,24,25,26,27,28,29,30,31,32,33,34,35,36,37,38,39,40,41,42,43,44,45,46,47,48,49]. The studies that were included in the analysis presented a low risk of bias in the AQUA score [50]. Furthermore, all of the articles achieved at least 30 points in the CATAM score, which should be interpreted as “Good” or “Very Good” quality. The complete procedure of the data collection is illustrated in Figure 3. Details regarding the studies included can be found in Table 1.

The mean diameter of the AEA in the intraorbital part was found to be 0.94 mm (SE = 0.05), whereas in the intracranial part, this was set to be 0.76 mm (SE = 0.03). The mean length of the AEA was set to be 4.64 mm (SE = 1.18). These aforementioned results are demonstrated in Table 2.

The relationship between the course of the AEA and the anterior skull base has been analyzed in three subgroups. In the overall analysis, reports of the AEA being embedded within the skull base or at the level of the skull base producing a bony protrusion were found to occur in 56.91% of the cases (95% CI: 43.55–69.80%). Moreover, in the analysis concerning only the computed tomography-based results, the pooled prevalence in this category was measured at 52.23% (95% CI: 36.43–67.81%), whereas in the analysis in which only results based on cadavers were evaluated, the pooled prevalence was established at 64.35% (95% CI: 37.00–87.85%). In the overall analysis, the case where the AEA courses freely in the ethmoid sinus within a bony anterior ethmoidal canal while connected to the skull base by a thin bony mesentery was found to occur in 43.09% of the results (95% CI: 30.20–56.45%). All of the aforementioned results are showcased in Table 3.

The most common localization of the AEA was found to be between the second and third lamellae, with the pooled prevalence of this being set at 65.82% (95% CI: 55.39–75.56%). Moreover, the AEA was found to occur between the first and second lamellae in 14.87% (95%CI: 0.00–38.08%) of cases. The mean distance between the AEA and the skull base was 1.76 mm (SE = 0.17). Furthermore, the mean distance between the AEA and the inferior turbinate was 30.86 mm (SE = 0.75). These abovementioned results are presented in Table 4.

Furthermore, an analysis of the outcomes with respect to the origin of the patients was performed. Among the categories meeting the asnalysis criteria, none of the results from various geographical locations showed statistically significant differences compared to the overall satisfactory outcome (*p* > 0.05).

## 4. Discussion

**Figure 4:**
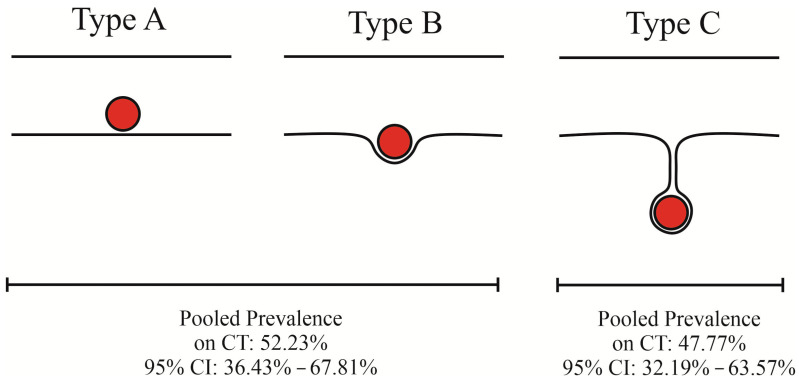
Different variations of the anterior ethmoidal artery’s course.

The AEA has received considerable attention due to its considerable variability and clinical significance. Numerous studies have investigated its location relative to various anatomical landmarks. As mentioned earlier, the location of the AEA with respect to the skull base has been described as either being in the skull base (Type A), at the skull base level and creating a bony protrusion (Type B), or having a free course in the ethmoid sinus while being connected to the skull base by a thin bony mesentery (Type C) [5,6,7]. Studies have reported varying frequencies for each type, with some indicating Type A as the most common, ranging from 57.0% to 85.7% of the cases [9,22]. However, other investigations have shown Type C to be the predominant course, with a frequency reaching 84.0% [11,23]. Despite this diversity in the reported frequencies, few studies have utilized the complete classification system separately, often grouping Types A and B together as indicative of the AEA being embedded in the skull base, with Type C representing the AEA coursing below the skull base. To mitigate potential bias and ensure data quality, the present meta-analysis treated Types A and B as a single category, while Type C was analyzed independently. Our findings revealed a strong similarity in prevalence between these courses, with Types A/B collectively representing the most frequent course with respect to the skull base, with a pooled prevalence of 56.91% (Figure 4). The discrepancies between the results in the literature have been stated to stem from methodological differences, particularly between cadaveric and CT-based studies [51]. To address this, our study performed a sub-analysis based on the type of subject sample used. Interestingly, Type A/B remained the most frequent type in both CT-based and cadaveric studies, with pooled prevalences of 52.23% and 64.35%, respectively. Nevertheless, these findings underscore a relatively even distribution of the AEA’s course between being embedded within the skull base and coursing freely below it. Therefore, prior to undertaking ESS or ESBS, it is imperative to conduct a CT scan to ascertain the course of the AEA and plan the procedure accordingly, thereby minimizing the risk of intraoperative complications.

The relationship between the anterior ethmoidal artery (AEA) and the four lamellae within the ethmoid sinus has garnered attention for its potential implications in aiding surgeons during intraoperative localization of this vessel. The lamellae are vertical bony structures that compartmentalize the ethmoid sinus and consist of the uncinate process (first lamella), ethmoid bulla (second lamella), basal lamella (third lamella), superior nasal concha (fourth lamella), and the anterior face of the sphenoid sinus (fifth lamella) [51]. Our study demonstrates that the most prevalent location of the AEA, in relation to the lamellae, is between the second and third lamellae (65.82%). However, our results showcase that this artery may also be located between the first and second lamella in relatively many cases (14.87%). Rarely, the AEA may extend beyond the third lamella (1.74%) or be localized within the posterior margin of the frontal sinus ostium (1.25%). This information holds significant value for surgeons, as inadvertent injury to the AEA may occur during the removal of lamellae during surgery. Additionally, our study examined the distances between the AEA and nearby anatomical landmarks. The mean distance between the vessel and the skull base was found to be 1.76 mm, highlighting its close proximity to this area. Furthermore, the distances between the AEA and the inferior and middle turbinates were measured at 30.86 mm and 20.98 mm, respectively. These data can undoubtedly aid in the intraoperative localization of this vessel during ESS and ESBS.

In recent years, purely endonasal endoscopic approaches for resecting anterior skull base malignancies have gained popularity, offering a minimally invasive alternative for surgical intervention [52]. Anterior skull base lesions, predominantly represented by highly vascularized meningiomas, pose a surgical challenge due to the risk of significant blood loss during resection [53]. Meningiomas situated in the olfactory groove and planum sphenoidale primarily obtain their blood supply from the dural branches originating from the internal carotid artery, which includes the AEA [53]. The meningeal branches of the AEA, including the anterior falcine artery, can become significantly enlarged in cases of meningiomas or other tumors in this region, complicating surgical resection due to the heightened risk of hemorrhagic complications [1]. To address this challenge, techniques for reducing blood loss during anterior skull base tumor resection have been explored, including preoperative direct ligation of the ethmoidal arteries [54]. Aref et al. [54] showed that ligating the ethmoidal artery could be advantageous in large-to-giant anterior skull base meningioma surgery. The findings of their study revealed a reduction in the average estimated blood loss and a lesser decline in pre- and postoperative hemoglobin and hematocrit levels in patients who underwent the ligation procedure compared to those who did not.

Furthermore, endoscopic ligation of the AEA has been utilized as a treatment for epistaxis refractory to nasal packing [42]. The intranasal approach offers advantages over external approaches, including no visible scars and reducing the risk of orbital complications. This procedure involves an anterior ethmoidectomy and exposure of the nasofrontal recess, followed by cauterization with bipolar electrocautery or placement of a hemoclip on the orbital side of the vessel. It is crucial to emphasize that the vessel is not transected to avoid retraction into the orbit, which may lead to orbital hematoma and subsequent vision impairment [55]. Comprehensive knowledge of the topographic characteristics of the AEA is crucial for surgeons to effectively localize this vessel and enhance operative safety during ESS and ESBS.

The current study has certain limitations that warrant acknowledgment. Potential biases could be present due to limitations in the accuracy of data gathered from diverse publications, thereby restricting the outcomes of this meta-analysis. Furthermore, the authors were unable to conduct certain analyses due to insufficient availability of consistent data. Data regarding the studied outcomes with respect to the sex of the patients’ are limited in the literature, and therefore, in order to provide the best possible quality of the results, we did not perform statistical analyses relating to the patients’ sex. However, we believe that such data should be studied in future research and such analyses should be performed in further meta-analyses. Additionally, data regarding the geographical origin of the studied patients were also limited in the literature. Therefore, some of the geographical analyses were not performed in order to prevent potential bias of the results. Such information should be included in further studies. Furthermore, due to heterogeneous classification systems, the extracted data had to be unified to provide new statistical outcomes. However, despite its limitations, our meta-analysis endeavors to delineate the anatomy of the AEA by utilizing data from the literature that aligns with the principles of evidence-based anatomy [56].

## 5. Conclusions

Different locations of the AEA with respect to the skull base (Type A/B and Type C) were reported with a relatively similar prevalence, and the artery coursed most commonly between the second and third lamellae. Comprehensive knowledge of the AEA’s topo-graphic characteristics is essential for optimizing surgical outcomes and ensuring patient safety in various sinus and skull base procedures. Our results may aid surgeons in effective intraoperative localization of this vessel, subsequently decreasing the potential risk of hemorrhagic complications. In conclusion, the AEA exhibits considerable anatomical variability and clinical significance, particularly in the context of ESS and ESBS.

## Figures and Tables

**Table 1:** Characteristics of the studies submitted to this meta-analysis. CT—computed tomography.

First Author	Year of Publication	Continent	Country	Method	Arteries Studied
Livashin Naidu [24]	2023	Africa	South Africa	CT + Cadavers	126
Nikma Fadlati Umar [26]	2022	Asia	Malaysia	CT	370
Melih Caklili [48]	2021	Asia	Turkey	Cadavers	6
Gian Luca Fadda [44]	2021	Europe	Italy	CT	440
Amr F. Hamour [40]	2020	North America	Canada	CT	60
Mustafa Cemil Kilinc [37]	2020	Asia	Turkey	Cadavers + CT	14
Mohamed A. Taha [28]	2020	North America	USA	CT	100
Teppei Takeda [27]	2020	Asia	Japan	CT	366
Mohammad Waheed El-Anwar [4]	2019	Africa/Asia	Egypt	CT	300
Baharudin Abdullah [51]	2018	Asia	Malaysia	CT	252
Jasmine P. Y. Kho [38]	2018	Asia	Malaysia	CT	108
Ana M. Lemos-Rodriguez [34]	2018	North America	USA	Cadavers	8
Marco Ferrari [43]	2017	Europe	Italy	Cadavers	28
Vinicius Tomadon Bortoli [33]	2017	South America	Brazil	CT	600
Eric Mason [32]	2015	North America	USA	CT	100
David W. Jang [39]	2014	North America	USA	CT	78
Young-Bum Ko [5]	2014	Asia	South Korea	CT	119
Yu Zong [25]	2014	Asia	China	Cadavers	16
Erdem Eren [45]	2013	Asia	Turkey	CT	298
Christian Güldner [41]	2011	Europe	Germany	CT	282
Francisco G. Pernas [31]	2011	North America	USA	CT	138
Ming Song [29]	2011	Asia	China	Cadavers	20
Anagha A. Joshi [20]	2010	Asia	India	CT	100
Bashar Abuzayed [49]	2009	Asia	Turkey	Cadavers	24
C. Arturo Solares [30]	2009	North America	USA	CT	8
You-xiong Yang [11]	2009	Asia	China	Cadavers	28
S. E. McDonald [21]	2008	Europe	United Kingdom	CT	42
Senem Erdogmus [46]	2006	Asia	Turkey	Cadavers	37
Bernardo Cunha Araujo Filho [8]	2006	South America	Brazil	Cadavers	48
Stephen R. Floreani [42]	2006	Australia	Australia	CT	44
D. Simmen [10]	2006	Europe	Switzerland	Cadavers	34
L. Lannoy-Penisson [6]	2006	Europe	France	Cadavers + CT	18
Cankal F. [23]	2004	Europe	Turkey	CT	150
Hyoung-Jin Moon [22]	2001	Asia	Korea	Cadavers + CT	70
Wai Chung Lee [35]	2000	Asia	China	Cadavers	56
Sema Basak [9]	1998	Asia	Turkey	CT	222
A Ducasse [47]	1985	Europe	France	Cadavers	70
John A. Kirchner [36]	1961	North America	USA	Cadavers	70

**Table 2:** Statistical results of this meta-analysis regarding the diameter and the length of the anterior ethmoidal artery (AEA).

Category	Mean	Standard Error	Variance	Lower Limit	Upper Limit	Z-Value	*p*-Value
Diameter							
Mean diameter of the AEA in the intraorbital part [mm]	0.94	0.05	0.00	0.85	1.04	19.52	0.00
Mean diameter of AEA in the intracranial part [mm]	0.76	0.03	0.00	0.70	0.81	28.44	0.00
Length							
Mean length of the AEA [mm]	4.64	1.18	1.40	2.32	6.96	3.91	0.00

**Table 3:** Statistical results of this meta-analysis regarding the pooled prevalence of each type of the anterior ethmoidal artery (AEA). LCI—lower confidence interval. HCI—higher confidence interval. Q—Cochran’s Q.

Category	Pooled Prevalence	LCI	HCI	Q	I^2^
Type of AEA according to its relationship with the anterior skull base					
AEA embedded within the skull base/AEA at the level of the skull base producing a bony protrusion (Type A/B) [Overall]	56.91%	43.55%	69.80%	217.47	94.94
AEA coursing freely in the ethmoid sinus within a bony anterior ethmoidal canal while connected to the skull base by a thin bony mesentery (Type C) [Overall]	43.09%	30.20%	56.45%	217.47	94.94
AEA embedded within the skull base/AEA at the level of the skull base producing a bony protrusion (Type A/B) [Computed Tomography]	52.23%	36.43%	67.81%	140.28	95.72
AEA coursing freely in the ethmoid sinus within a bony anterior ethmoidal canal while connected to the skull base by a thin bony mesentery (Type C) [Computed Tomography]	47.77%	32.19%	63.57%	140.28	95.72
AEA embedded within the skull base/AEA at the level of the skull base producing a bony protrusion (Type A/B) [Cadavers]	64.35%	37.00%	87.85%	53.84	92.57
AEA coursing freely in the ethmoid sinus within a bony anterior ethmoidal canal while connected to the skull base by a thin bony mesentery (Type C) [Cadavers]	35.65%	12.15%	63.00%	53.84	92.57

**Table 4:** Statistical results of this meta-analysis regarding the topographical location of the anterior ethmoidal artery (AEA). LCI—lower confidence interval. HCI—higher confidence interval. Q—Cochran’s Q.

Category	Pooled Prevalence	LCI	HCI	Q		I^2^	
Localization of the AEA							
AEA between the second and third lamellae	65.82%	55.39%	75.56%	7.70		87.01	
AEA between the first and second lamellae	14.87%	0.00%	38.08%	53.17		98.12	
AEA in the second lamella	4.95%	0.00%	30.07%	100.78		99.01	
AEA in the third lamella	4.09%	0.00%	24.31%	80.51		98.76	
AEA beyond the third lamella	1.74%	0.49%	3.64%	2.31		56.78	
AEA within the posterior margin of the frontal sinus ostium	1.25%	0.00%	3.35%	4.45		77.52	
**Category**	**Mean**	**Standard Error**	**Variance**	**Lower Limit**	**Upper Limit**	**Z-Value**	***p*** **-Value**
Distance between the AEA and nearby anatomical landmarks							
Distance between AEA and skull base [mm]	1.76	0.17	0.03	1.43	2.09	10.42	0.00
Distance between AEA and inferior turbinate [mm]	30.86	0.75	0.56	29.39	32.33	41.14	0.00
Distance between AEA and middle turbinate [mm]	20.98	0.27	0.07	20.45	21.50	78.50	0.00
Distance between AEA and columella [mm]	63.56	0.41	0.17	62.76	64.36	155.14	0.00
Distance between AEA and nasofrontal beak [mm]	11.60	5.26	27.68	1.29	21.91	2.20	0.03

## Data Availability

The data that support the findings of this study are available from the corresponding authors, upon reasonable request.
